# Sarcopenia is associated with non-motor symptoms in Han Chinese patients with Parkinson's Disease: a cross-sectional study

**DOI:** 10.1186/s12877-023-04188-3

**Published:** 2023-08-16

**Authors:** Qiu-Wan Liu, Cheng-Jie Mao, Zhao-Hui Lu, Rong-Fang Shi, Ying-Chun Zhang, Ping Zhao, Chun-Feng Liu

**Affiliations:** 1 Department of Neurology and Clinical Research Center of Neurological Disease, The Second Affiliated Hospital of Soochow University, Suzhou, 215004 China; 2 Department of Neurology, The Second People’s Hospital of Hefei, Hefei Hospital Affiliated to Anhui Medical University, Hefei, Anhui 230011 China; 3 Institute of Neuroscience, Soochow University, Suzhou, 215004 China

**Keywords:** Parkinson’s disease, Sarcopenia, Fatigue, Sleep quality, Older adults, Non-motor symptoms

## Abstract

**Background:**

Sarcopenia is commonly seen in the older adults and increases in incidence with age, also in Parkinson’s disease (PD). Although research has indicated that the development of sarcopenia in patients with PD may be related to both motor symptoms and non-motor symptoms (NMS), the precise relationship between the two conditions remains unclear. Therefore, we aimed to investigate the incidence of sarcopenia in patients with PD and its association with NMS.

**Methods:**

The study included 123 patients with PD and 38 age- and sex-matched healthy controls (HC). All participants were evaluated for sarcopenia using the 2019 Asian Sarcopenia Diagnostic Criteria, and patients with PD underwent standard assessments of motor symptoms and NMS. Multiple logistic regression and receiver operating characteristic (ROC) curve analyses were used to examine the association between sarcopenia and NMS in patients with PD.

**Results:**

The incidence of sarcopenia was significantly higher in patients with PD than in HC (26.8% vs. 10.4%, *p* = 0.046). Multiple logistic regression analysis revealed that poorer sleep quality (odds ratio [OR]: 1.245; 95% confidence interval [CI]: 1.011–1.533; *p* = 0.040) and fatigue (OR: 1.085, 95% CI: 1.006–1.170, *p* = 0.034) were independently associated with sarcopenia. ROC analysis indicated that the optimal cut-off value for Pittsburgh Sleep Quality Index (PSQI) scores was 10, with 72.7% sensitivity and 74.4% specificity (area under the curve [AUC] = 0.776, 95% CI: 0.683–0.868, *p* < 0.001). The optimal cut-off value for Fatigue Severity Scale (FSS) scores was 39, with 87% sensitivity and 50% specificity (AUC = 0.725, 95% CI: 0.629 –0.820, *p* < 0.001). Joint use of FSS and PSQI scores increased the predictive value for sarcopenia(AUC = 0.804, 95% CI: 0.724–0.885, *p* < 0.001).

**Conclusion:**

Patients with PD are more susceptible to sarcopenia than healthy older adults, and fatigue and poorer sleep are positively associated with sarcopenia. Further longitudinal studies are needed to clarify the causal relationships.

## Background

Parkinson’s disease (PD) represents the second most common neurodegenerative disease worldwide and is among the conditions exhibiting the fastest increases in morbidity, disability, and mortality [1]. The development of PD is considered to be closely related to aging [2]. According to Chinese epidemiological data, the incidence of PD in individuals over 65 years old is 1.7%. The disease is mainly characterised by static tremor, bradykinesia, muscle rigidity, and postural balance disorders, although it is accompanied by various non-motor symptoms(NMS) including sleep disorders, cognitive dysfunction, autonomic abnormalities, mood disturbances, and pain. These NMS occur 60–100% of patients with PD and may play a greater impact on quality of life than motor symptoms [3, 4].

Sarcopenia is an age-related skeletal muscle pathology characterised by reduced muscle mass and muscle strength accompanied by a decline in physical performance decline. Although it has something with the aging process, sarcopenia is more commonly observed in older adults with chronic diseases such as Parkinson’s syndrome [5–8]. Indeed, previous studies have demonstrated that the prevalence of sarcopenia was significantly higher in patients with PD than in the older adults [9]. Sarcopenia has recently been classified as a geriatric syndrome [10] and is closely related to reduced quality of life, impaired cardiopulmonary function [11], falls, and fractures [12]. These in turn lead to increases in the frequency of emergency visits, hospitalization, and mortality in older adults [13].

Studies have indicated that the development of sarcopenia in patients with PD may be related to both motor symptoms and NMS [14]. Several groups have reported that sarcopenia is associated with disease duration and stage, as well as Unified Parkinson’s Disease Rating Scale (UPDRS)-I/II scores, depression, and cognitive function [9, 14–16]. However, other studies have reported controversial results [17], and there are large differences in the incidence of sarcopenia in different countries [18, 19]. Furthermore, to our knowledge, few studies have investigated sarcopenia in Asian populations, and none have reported sarcopenia data for patients with PD in China.

Therefore, in the present study, we investigated the incidence of sarcopenia in patients with PD as well as the association between NMS and PD risk using 2019 Asian Sarcopenia Diagnostic Criteria. Our findings may provide new insight into the importance of sarcopenia in the context of PD, which is essential for improving prognosis in this population.

### Methods

## Study participants

A retrospective cross-sectional design was carried out in the Chinese Han population, and a total of 140 patients with PD and 38 healthy controls (HC) were enrolled. Patients with PD were recruited from the neurology ward and outpatient clinic of the Second Affiliated Hospital of Soochow University from October 2020 to April 2021. The inclusion criteria were as follows: (1) the diagnosis of PD in accordance with the 2016 Chinese Parkinson's Disease Diagnostic Criteria; (2) willingness to undergo assessments with each scale. Patients meeting the following criteria were excluded from the study: (1) Parkinson’s syndrome or Parkinson’s superimposed syndrome stemmed from encephalitis, cerebrovascular disease, poisoning, trauma, drugs or other factors; (2) prior treatment with deep brain electrical stimulation; (3) diagnosis of severe anxiety, depression, schizophrenia, cancer, or physical disease; (4) had contraindications to bioelectrical impedance analysis (i.e. implanted medical devices). HC were recruited from the physical examination center during the same period. HC without a family history of PD or other neurodegenerative diseases, with no history of losing more than 10% of body weight within the last 6 months. HC were matched for age and gender with patients with PD. All participants should provide written informed consent in person, and the study won the approval of the Ethics Review Committee of The Second Affiliated Hospital of Soochow University. All procedures were conducted by following the guidelines of the 1964 Helsinki Declaration.

### Data collection

Demographic information was gathered for all study participants,including age, sex, height, education background, status of nutrition, past medical history and history of smoking and drinking tea or coffee. In our study, we recorded the education background according to their years of education. Mini nutritional assessment(MNA) scale were used to assess the nutritional status of patients. Screening scores > 12 or an overall score of ≥ 24 is defined as no risk of malnutrition, while total scores < 24 were defined as risk of malnutrition.

Additional data collected for patients with PD were evaluated during the "on" period included disease course, Hoehn and Yahr stage (H&Y), UPDRS scores, and daily Levodopa Equivalent Dose (LED). The motor symptoms were divided into tremor type, postural instability and gait difficulty (PIGD) type and mixed type. According to HY stages, it can be divided into early (HY ≤ 2.5 stage) and middle-late (HY > 2.5 stage). Dyskinesia and motor fluctuations (MF) were recorded based on part four of the UPDRS. Moreover, the Non-Motor Symptom Questionnaire (NMSQ) was used to screen for salivation, hyposmia, constipation, and frequent urination. The Hamilton Depression Scale (HAMD) and Hamilton Anxiety Scale (HAMA) scaled the depression and anxiety. The Epworth Sleepiness Scale (ESS), Pittsburgh Sleep Quality Index (PSQI), and Parkinson's Disease Quality of Life Scale-8 (PDQ-8) were used to evaluate subjective sleepiness, sleep quality, and quality of life, respectively. Fatigue, autonomic dysfunction, ability to perform activities of daily living, and cognitive function were assessed using the Fatigue Severity Scale (FSS), Scales for Outcomes in Parkinson's Disease-Autonomic Dysfunction (SCOPA-AUT), Ability of daily living scale (ADLs), and the Mini Mental State Examination (MMSE), respectively.

### Diagnosis of sarcopenia

Patients exhibiting decreased muscle strength or physical function combined with decreased muscle mass were diagnosed with sarcopenia, in accordance with the Asian Working Group for Sarcopenia 2019 Consensus on the Diagnosis and Treatment of Sarcopenia [20].

### Muscle strength

Grip strength was measured using a Camry Hand Dynamometer (Guangdong Xiangshan Weighing Apparatus, Zhongshan City, Guangdong Province). Measurements were obtained with the patient in the standing position and with the elbow in extension. Maximum isometric strength was measured for the dominant hand or both hands, and the largest reading after two tests was selected. Low muscle strength was defined as < 28.0 kg in men and < 18.0 kg in women.

### Physical function

Physical function was assessed via a 6-m gait speed (GS) test. The speed during the time required to walk 6 m at a normal gait speed was recorded from the beginning of movement, without considering acceleration or deceleration. Two tests were performed, and the average value was used for analysis. A pace of ≥ 6 s was considered to reflect decreased physical function.

### Muscle mass

Body composition was assessed via multi-frequency bioelectrical impedance analysis (BIA) (InBody720, Korea), which was considered in conjunction with height to determine each patient’s appendicular skeletal mass (ASM). All patients underwent assessment after fasting and excretion in the morning. Low muscle mass was defined as < 7.0 kg/m^2^ in men and < 5.7 kg/m^2^ in women.

### Statistical analysis

All statistical analyses were showed by the Statistical Package for the Social Sciences version 24.0 (SPSS Co., Chicago, IL, USA). Measurement data conforming to the normal distribution are showed as the mean ± standard deviation (SD), while those with a non-normal distribution as the median (interquartile range). Categorical parameters were expressed using frequencies and percentages. Differences in continuous variables among study groups came to assess using Student’s t-test or the Mann-Whitney U-test, while differences in the distribution of categorical variables taking use of the chi-square test or Fisher’s exact test. Univariate and multivariate logistic regression analysis was performed to examine the relationship between NMS and sarcopenia. Receiver operating characteristic (ROC) analysis was performed in order to investigate the diagnostic value about sleep quality and fatigue severity in the identification of sarcopenia. Statistical power was calculated by comparing frequencies or means of PD with sarcopenia and PD without sarcopenia in the PASS 11.0 software. A *p*-value of < 0.05 was treated statistically significant.

## Results

### Clinical and demographic data

**Fig. 1:**
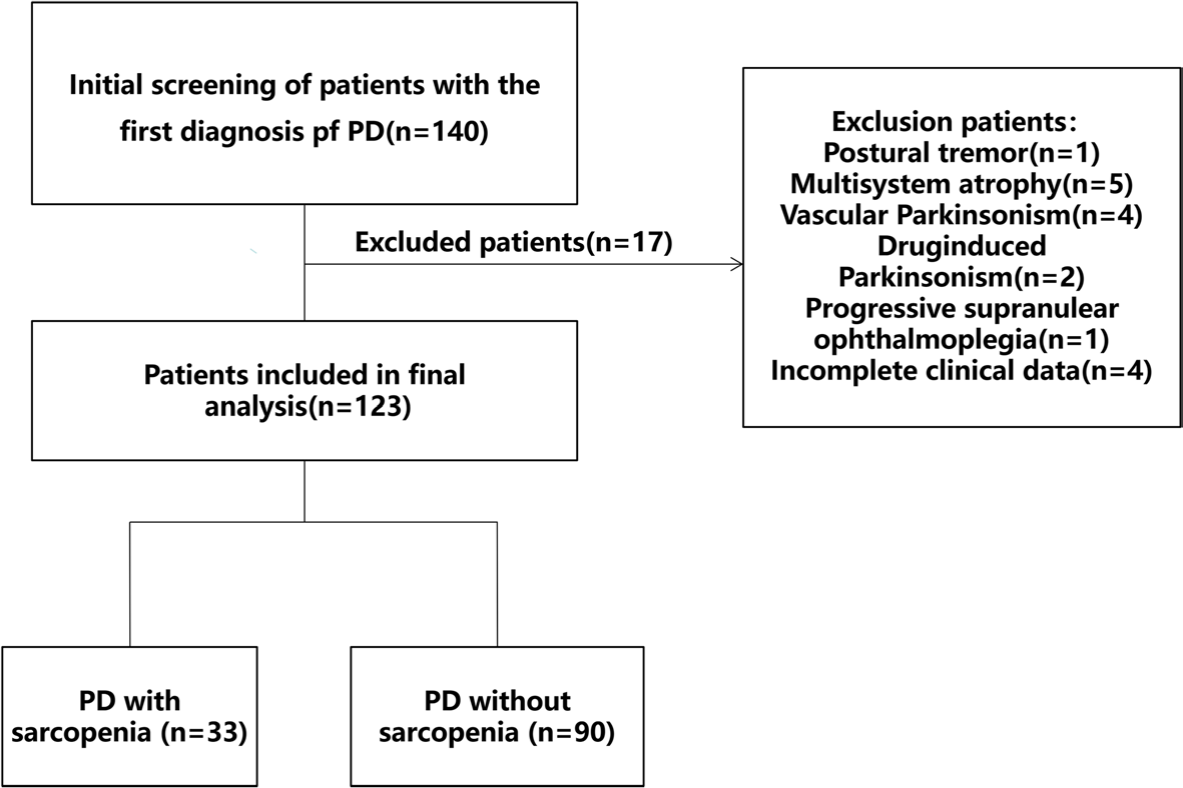
Flow diagram of the study participants

A total of 140 patients with PD and 38 HC were enrolled in our study, and 123 patients were included in the final analysis (Fig. 1). All participants were of Han Chinese descent. The PD group included 56 men and 67 women. Average ages in the PD and HC group were 65.73 ± 9.36 years and 67.03 ± 10.89 years, respectively. Sarcopenia was observed in 33 (26.8%) patients with PD and 4 (10.4%) control participants, and the incidence of sarcopenia was significantly higher in the PD group than in the HC group (*p* = 0.046). The prevalence of sarcopenia in the HC group was 0.105 (95% CI: 0.003–0.207), while that in the PD group was 0.268 (95% CI: 0.189–0.384). Body mass index (BMI) (*p* = 0.009) and grip strength (*p* < 0.001) were lower in patients with PD than in HC, although there were no significant differences in ASM(*p* = 0.075) and nutritional status(*p* = 0.150) between the two groups. GS was also slower in the PD group than in the HC group (*p* = 0.021). The comparison of demographic characteristics and sarcopenia parameters is presented in Table 1.

**Table 1:** Demographic characteristics and sarcopenia parameters of patients with PD and HC group

Variable	HC(*n* = 38)	PD(*n* = 123)	t/x^2^/z	*P*-Value
Age, y	67.03 ± 10.89	65.73 ± 9.36	0.716	0.475
Sarcopenia, n(%)	4(10.5)	33(26.8)		**0.046**
Low muscle mass, n(%)	4(10.5)	33(26.8)		**0.046**
Low muscle strength, n(%)	14(36.8)	48(39.0)	0.058	0.809
6 m GS ≥ 6 s, n(%)	22(57.9)	101(82.1)	9.444	**0.002**
Education,y	6.5(5,9)	7(4,9)	-0.292	0.770
Male, n(%)	23(60.5)	56(45.5)	2.613	0.106
Hypertension, n(%)	9(23.7)	34(27.7)	0.232	0.630
Type 2 diabetes, n(%)	5(13.2)	13(10.6)	0.196	0.658
Smoker, n(%)	6(15.8)	24(19.5)	0.265	0.606
Alcohol user, n(%)	7(18.4)	21(17.1)	0.037	0.848
Tea or coffee user, n(%)	10(26.3)	33(26.8)	0.004	0.950
state of nutrition			2.076	0.150
No risk of malnutrition, n(%)	23(60.5)	58(47.2)		
Risk of malnutrition, n(%)	15(39.5)	65(52.8)		
BMI (kg/m^2^)	25.19 ± 2.58	23.64 ± 3.32	2.638	**0.009**
ASM (kg/m^2^)	7.24(6.57,7.86)	6.83(5.91,7.70)	-1.178	**0.075**
Grip strength (kg)	25.35(20.65,33.6)	22.60(18.05,30.55)	-2.307	**0.021**
6 m GS (s)	6.02(5.36,6.83)	7.26(6.41,8.48)	-4.459	** < 0.001**

Values in bold are significant at *P* < 0.05

Data were expressed as n (%), mean ± SD, median (interquartile range). *P* values are from Student’s t-test or the Mann–Whitney U-test or Chi-square tests or Fisher’s exact test

Abbreviations: *PD* Parkinson's disease, *HC* healthy controls, *BMI* body mass index, *ASM* appendicular skeletal mass, *6 m GS* 6-m gait speed

### Clinical features of PD with and without sarcopenia

A total of 33 participants in the PD group had sarcopenia, while the remaining 90 did not. The demographic and clinical characteristics of patients with PD with and without sarcopenia are summarised in Table 2. The sarcopenia group included 11 men and 22 women, although the difference in gender was not significant (*p* = 0.1). Sarcopenia was more common in patients with advanced PD (*p* = 0.041) but was not correlated with disease duration, motor symptoms, or LED. Our analysis also indicated that patients with PD who had sarcopenia were older and had poorer nutritional status and ADL function, lower BMI, higher UPDRS scores (parts I, II, and IV), and poorer quality of life than those with PD who did not have sarcopenia. In addition, we observed significantly higher NMS burden in the sarcopenia group.

**Table 2:** Comparison of basic clinical data, motor symptoms and NMS of PD patients with or without sarcopenia

Variable	Without Sarcopenia	With Sarcopenia	t/x^2^/z	*P*-Value	Power (%)
	(*n* = 90)	(*n* = 33)			
Age,y	63.37 ± 8.99	72.18 ± 7.12	-4.209	** < 0.001**	10.0
Disease duration,y	4(2,7)	6(3,9.5)	-1.772	0.076	50.6
Education background,y	7.5(4.75,9)	6(2,9.5)	-0.460	0.646	37.5
Male, n(%)	45(50)	11(33.3)	2.705	0.100	37.6
Hypertension, n(%)	27(30)	7(21.2)	0.932	0.334	16.2
Type 2 diabetes, n(%)	11(12.2)	2(6.1)		0.551	16.6
Smoker, n(%)	20(22.2)	4(12.1)	1.569	0.210	24.0
Alcohol user, n(%)	17(18.9)	4(12.1)	0.781	0.377	14.3
Tea or coffee user, n(%)	25(27.8)	8(24.2)	0.154	0.695	6.7
Types of Motor Symptoms			0.795	0.672	11.5
Tremor-dominant, n(%)	28(31.1)	8(24.2)			
PIGD-dominant, n(%)	50(55.6)	19(57.6)			
Mixed, n(%)	12(13.3)	6(18.2)			
state of nutrition			7.291	**0.007**	97.4
no risk of malnutrition, n(%)	52(57.8)	6(18.2)			
Risk of malnutrition, n(%)	38(42.2)	27(81.8)			
BMI (kg/m^2^)	24.74 ± 2.72	20.64 ± 2.96	7.225	** < 0.001**	100
ASM (kg/m^2^)	7.36(6.38,7.89)	5.5(5.18,6.66)	-6.336	** < 0.001**	100
Grip strength (kg)	24.9(18.7,32.63)	18.7(15.3,22.05)	-4.167	** < 0.001**	73
6 m GS (s)	7.04(5.99,8.33)	7.75(7.15,9.43)	-2.934	**0.003**	36.6
UPDRS-I	3(2,5)	4(2.5,6)	-2.056	**0.040**	39.8
UPDRS-II	11(8,16.25)	15(9,22.5)	-2.501	**0.012**	59.1
UPDRS-III	24(15,33)	26(13.5,33)	-0.231	0.817	8.9
UPDRS-IV	1(0,3)	2(0.5,6)	-2.793	**0.005**	29.8
Dyskinesia, n(%)	11(12.2)	6(18.2)	0.72	0.396	13.6
MF, n(%)	20(22.2)	13(39.4)	3.627	0.057	47.8
H-Y early stage, n(%)	71(78.9)	20(60.6)	4.193	**0.041**	53.5
LED (mg/d)	387.5(225, 654.75)	499(293.75,724)	-1.369	0.171	28.1
MMSE	26(23,28)	23(19.5,27)	-2.446	**0.014**	84.2
PSQI	8(5.75,11)	13(10,15.5)	-4.693	** < 0.001**	99.2
ESS	4(3,9)	7(3,12.5)	-1.891	0.059	63.0
FSS	39.5(27.75,48.25)	49(43.5,53.5)	-3.815	** < 0.001**	75.0
HAMA	8(5,13)	16(10.5,21)	-4.328	** < 0.001**	99.9
HAMD	9(6,13.25)	17(12.5,22.5)	-4.157	** < 0.001**	99.9
NMSQ	8(5,12.25)	12(8,17)	-3.383	**0.001**	78.9
SCOPA-AUT	10(6,14)	15(10.5, 19.5)	-3.297	**0.001**	89.6
PDQ-8	8.5 ± 4.81	12.39 ± 5.57	-3.81	** < 0.001**	98.5
ADLs	24(21,35)	37(25.5,56)	-3.753	** < 0.001**	99.9

Values in bold are significant at *P* < 0.05

Data were expressed as n (%), mean ± SD, median (interquartile range). *P* values are from Student’ s t-test or the Mann–Whitney U-test or Chi-square tests or Fisher’s exact test

Abbreviations: *PD* Parkinson's disease, *PIGD* postural instability and gait difficulty, *BMI* Body Mass Index, *ASM* appendicular skeletal mass, *6 m GS* 6-m gait speed, *UPDRS* Unified Parkinson's Disease Rating Scale, *MF* motor fluctuations, *LED* Levodopa Equivalent Dose, *MMSE* Mini Mental State Scale, *PSQI* Pittsburgh Sleep Quality Index, *ESS* Epworth Sleep Scale, *FSS* Fatigue Severity Scale, *HAMA* Hamilton Anxiety Scale, *HAMD* Hamilton Depression Scale, *NMSQ* Non-Motor Symptom Questionnaire, *SCOPA-AUT* Scales for Outcomes in Parkinson's Disease-Autonomic questionnaire, *PDQ-8* Parkinson's Disease Quality of Life Scale-8, *ADLs* Ability of Daily Living scale

### Univariate and multivariate logistic regression analysis of risk factors for PD with sarcopenia

In order to further determine whether the NMS of PD patients are related to the occurrence of sarcopenia, univariate and multivariate logistic regression models was used in this study to analyze the correlation between baseline clinical characteristics and the occurrence of sarcopenia in PD patients. According to previous studies, age and low BMI are independent risk factors for sarcopenia. Considering the small sample size of our study and a large number of independent variables, we adjusted for recognized potential confounders such as age and BMI in univariate logistic regression. The results showed that the nutritional status, fatigue and sleep quality of PD patients were closely related to the occurrence of sarcopenia (OR values were 5.477, 1.078, 1.292, *p* < 0.05). (Table 3).

**Table 3:** Univariate logistic regression analysis of sarcopenia in PD patients

Univariate analysis	Model 1	Model 2
	OR(95% CI)	OR(95% CI)
MMSE	0.920(0.856,0.989)	0.964(0.872,1.067)
FSS	1.077(1.033,1.123)	**1.078(1.012,1.148)**
PSQI	1.253(1.130,1.390)	**1.292(1.093,1.527)**
HAMA	1.102(1.042,1.164)	1.080(0.999,1.166)
HAMD	1.097(1.042,1.154)	1.068(0.997,1.143)
NMSQ	1.104(1.023,1.193)	1.103(0.984,1.236)
SCOPA-AUT	1.104(1.036,1.177)	1.086(0.992,1.118)
UPDRS I	1.233(1.028,1.479)	0.999(0.777,1.284)
UPDRS II	1.086(1.026,1.149)	1.107(0.945,1.095)
UPDRS IV	1.200(1.039,1.386)	1.191(0.964,1.672)
HYstage	2.429(1.025,5.754)	0.922(0.261,3.251)
state of nutrition	0.318(0.136,0.745)	**5.477(1.125,26.665)**

Model 1: Univariate logistic regression analysis unadjusted; Model 2: Univariate logistic regression analysis adjusted for age (in years, SD corrected), BMI

Abbreviations: *MMSE* Mini Mental State Scale, *FSS* Fatigue Severity Scale, *PSQI* Pittsburgh Sleep Quality Index, *HAMA* Hamilton Anxiety Scale, *HAMD* Hamilton Depression Scale, *NMSQ* Non-Motor Symptom Questionnaire, *SCOPA-AUT* Scales for Outcomes in Parkinson's Disease-Autonomic questionnaire, *BMI* body mass index

In addition, although gender did not show a statistically significant difference between the two groups (*p* = 0.1). Considering the differences in muscle mass and strength between men and women in general, the probability of developing sarcopenia in women (32.8%) in this study was significantly higher than that in men (19.6%), thus gender was also included in the multi-factor logistic regression model for analysis, and the Enter regression method was adopted. After adjusting for age, BMI, nutritional status, and gender, multiple logistic regression analysis showed that poorer sleep quality (OR: 1.245; 95%CI: 1.011–1.533; *p* = 0.040) and fatigue (OR: 1.085, 95% CI: 1.006–1.170, *p* = 0.034) were independently associated with sarcopenia. (Table 4).

**Table 4:** Multivariate logistic regression analysis of sarcopenia in PD patients

Variable	OR	95%CI	*P*-Value
FSS	1.085	1.006 ~ 1.170	**0.034**
PSQI	1.245	1.011 ~ 1.533	**0.040**
state of nutrition	9.671	1.355 ~ 69.018	0.024
Age	1.212	1.091 ~ 1.346	< 0.001
BMI	0.401	0.256 ~ 0.629	< 0.001
gender	1.292	0.298 ~ 5.600	0.732

Abbreviations: *FSS* Fatigue Severity Scale, *PSQI* Pittsburgh Sleep Quality Index, *BMI* body mass index

### Optimal cut-off value for discriminating sarcopenia in PD

**Fig. 2:**
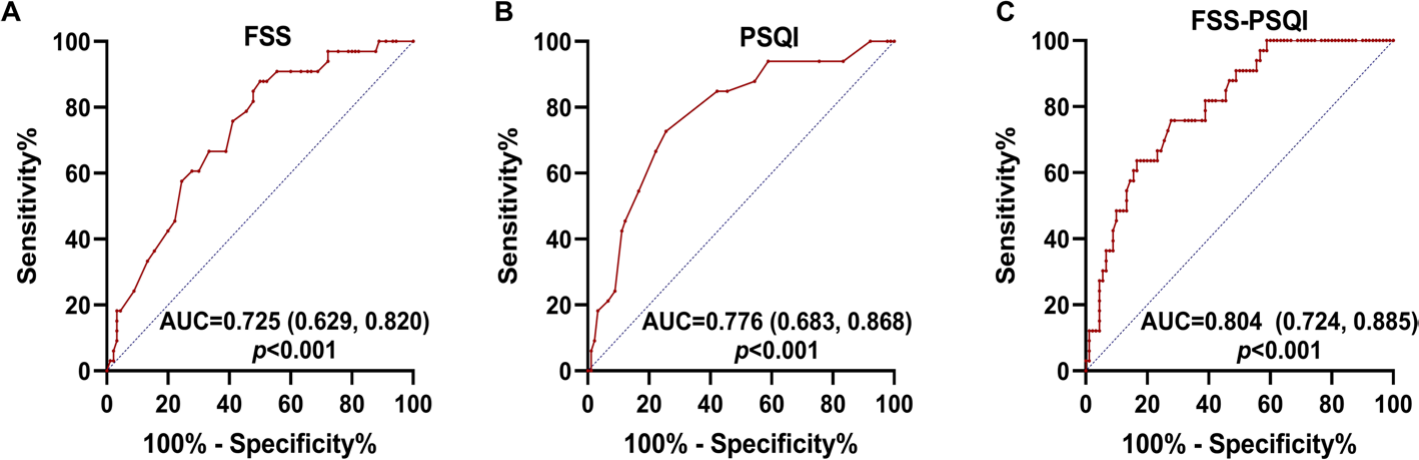
ROC showed the best cut-off value of fatigue severity score, PSQI score and the combined group to distinguish sarcopenia in PD

ROC curve analysis was used to evaluate the potential ability of NMS to distinguish sarcopenia in PD. The area under the curve (AUC) values were 0.725 (95% CI: 0.629–0.820; *p* < 0.001) for the FSS score and 0.776 (95% CI: 0.683- 0.868; *p* < 0.001) for the PSQI score. Further analyses of diagnostic performance revealed that the FSS could differentiate sarcopenia in PD with a sensitivity of 87% and a specificity of 50%, while the sensitivity and specificity values for the PSQI were 72.7% and 74.4% respectively (Fig. 2). However, joint use of FSS and PSQI increased the predictive value for sarcopenia (AUC = 0.804, 95% CI: 0.724–0.885; *p* < 0.001) (Fig. 2). A trend significant differences between the FSS group and the combined group (*p* = 0.0535),whereas there was no significant difference between the PSQI group and FSS-PSQI group(*p* = 0.3473).

## Discussion

To date, few studies have investigated sarcopenia in patients with PD, especially within the Han population. This is the first study to report that sarcopenia is strongly and independently associated with symptoms of fatigue and severe disturbances in sleep quality in Han Chinese patients with PD. Our results also indicated that the incidence of sarcopenia was greatly higher in patients with PD than in HC, which is accordance with the results reported in previous studies [9]. Furthermore, our ROC analysis indicated that combined use of FSS and PSQI scores may be a valuable screening method for identifying sarcopenia in patients with PD.

Our analysis confirmed that the appearance of sarcopenia is significantly associated with the age of the patient, which is consistent with the definition of sarcopenia as a geriatric disease and published geriatric literature [21, 22]. Previous studies have demonstrated that older adults often experience a reduction in skeletal muscle mass, which is considered to be related to nervous system alterations and changes in metabolism, hormone levels, nutrition, and physical activity [22, 23]. These changes in muscle mass and function can lead to disability, reduced mobility, functional dependence, and functional decline [24–26]. Similarly, in our study, patients with PD who had sarcopenia exhibited poorer nutritional status and lower BMI than their counterparts without sarcopenia. From a clinical perspective, these results highlight the need to screen for sarcopenia in older adults with PD and address changes in nutritional status and BMI as early as possible in the treatment process.

Notably, although many patients with PD in our study were in the early stage of the disease, a loss of muscle mass was still observed in these patients. Furthermore, in accordance with the findings of Tamer et al. [27], we observed significant decreases in muscle mass with disease progression. These decreases in muscle mass exert a negative impact on patient prognosis, emphasizing the importance of early detection and intervention.

The incidence of sarcopenia was also higher in our sample of patients with PD than in the general older adult population in China [28]. This may be related to the existence of common pathophysiological pathways affected by PD and sarcopenia, including those associated with inflammation, autophagy, oxidative stress, and apoptosis [16, 29–32]. Changes in brain structure and brain networks are thought to exert a substantial influence on the pathophysiology of sarcopenia in patients with PD [33]. In addition, Drey et al. [16] reported that the UPDRS-III score is significantly related to early sarcopenia, indicating that a common pathway may be involved in the preclinical stage of the two diseases, and that sarcopenia may be a sign of PD in the preclinical stage.The number of motor neurons decreased in elderly patients with sarcopenia and PD [34, 35], suggesting that neurodegeneration plays a key role in the pathogenesis of sarcopenia. Sarcopenia may also be affected by hormonal changes and medications in patients with PD [36, 37]. In addition, PD-related motor dysfunction may lead to a significant decline in muscle mass and physical ability relative to the level of healthy older adults [27].

Our findings indicated that sarcopenia as determined based on BIA findings is negatively associated with sleep quality and the severity of fatigue in patients with PD. Sleep and fatigue are the two main NMS of PD. Further, the prevalence of sleep disorders in patients with PD is 47.7-89.1%, which is higher than that in the general population [38]. Sleep disorders in patients with PD may be due to degeneration of thalamocortical pathways and alterations in neurotransmitter levels [39]. Recent research has indicated that sleep may exert an important influence on muscle protein metabolism [40]. In addition, studies have suggested that levels of growth hormone (GH), insulin-like growth factor-1 (IGF-1), and testosterone play a role in the association between sarcopenia and sleep disorders [41].

Fatigue refers to the subjective experience of a lack of energy and physical exhaustion, which is common in patients with PD [42] and can bring about the quality of life go down. Studies have also reported a close relevance between muscle mass and fatigue in patients with cancer [43], suggesting that decreased muscle mass is a critical factor in the development of fatigue. Given that sleep and fatigue may be involved in the association between PD and sarcopenia, we attempted to determine whether FSS and PSQI scores had enough discriminative power to distinguish sarcopenia in patients with PD. ROC curve analysis revealed that the AUC values for the FSS and PSQI were 0.725 and 0.776, respectively, while that for combined use of the FSS and PSQI reached 0.804. These results indicate that FSS and PSQI scores exhibit acceptable sensitivity and specificity for the potential discrimination of sarcopenia in patients with PD.

Our study demonstrates the incidence of sarcopenia is higher in PD patients than healthy. The occurrence of sarcopenia in PD patients is closely related to NMS, especially fatigue and poorer sleep. Attention and possible intervention measures for fatigue and poorer sleep may be helpful for avoiding sarcopenia in patients with PD. In addition, this study provides information from mainland China for research on sarcopenia in PD patients.

The present study had some limitations. First, the study was executed at the PD Clinic in Suzhou, where eating habits and ethnicity are relatively homogenous. Therefore, our findings may not be applicable to other, more diverse populations, highlighting the need for multicentre studies to confirm our results. Second, in the early stages of sarcopenia, the loss of muscle mass in the lower limbs may precede the loss of muscle mass in the upper limbs, so considering the loss of total muscle mass alone may not be sufficient to account for muscle loss in a timely manner, as there may be normal or even compensatory increases in muscle mass in the upper limbs that affect the assessment. In this sense, considering total muscle mass rather than local loss to determine the diagnosis of sarcopenia may actually lead to misdiagnosis, and we excluded patients who were unable to cooperate with the assessment and patients with advanced PD who were unable to stand independently, which may have led us to further underestimate the prevalence of sarcopenia. Because these excluded patients may have more severe features than those included. Article by Kara M [44] presents a new diagnostic algorithm suggesting that the use of sonographic thigh adjustment ratio (STAR) is more valuable than early assessment and timely diagnosis of low muscle mass than ASM. Recent cross-sectional studies have also reported that muscle strength and performance tests correlate better with thigh anterior muscle thickness than total muscle mass measures [45, 46]. Therefore, in future longitudinal studies, it would undoubtedly be more appropriate to normalize the data and associated morbidity and mortality projections using validated STAR cutoff values from one's own national standards. In addition, we did not use objective tools for assessments of sleep. In future studies, polysomnography (PSG) can be used to better evaluate and analyse the relationship between sleep characteristics and sarcopenia in patients with PD. Finally, the cross-sectional nature and small sample size of this study only allow for a preliminarily report on the incidence of sarcopenia in patients with PD and its relationships with fatigue and sleep. Thus, further studies with long-term follow up and large sample sizes are required to determine the causal direction of these relationships.

## Conclusion

The present findings demonstrate that the prevalence of sarcopenia is significantly higher among patients with PD than among general population in China. Moreover, in the PD group, patients with sarcopenia had worse sleep quality and more severe fatigue than those without. Therefore, interventions for addressing decreases in muscle mass in the early stages of PD are urgently required.

## Data Availability

The datasets used and analysed during the current study are available from the corresponding author on reasonable request.

## Abbreviations

PD: Parkinson’s disease
HC: healthy controls
NMS: non-motor symptoms
ROC: receiver operating characteristic
OR: odds ratio
CI: confidence interval
PSQI: Pittsburgh Sleep Quality Index
AUC: area under the curve
FSS: Fatigue Severity Scale
UPDRS: Unified Parkinson’s Disease Rating Scale
MNA: mini nutritional assessment
H&Y: Hoehn and Yahr stage
LED: Levodopa Equivalent Dose
PIGD: postural instability and gait difficulty
MF: motor fluctuations
NMSQ: Non-Motor Symptom Questionnaire
HAMD: Hamilton Depression Scale
HAMA: Hamilton Anxiety Scale
ESS: Epworth Sleepiness Scale
PDQ-8: Parkinson's Disease Quality of Life Scale-8
SCOPA-AUT: Scales for Outcomes in Parkinson's Disease-Autonomic Dysfunction
ADLs: Ability of daily living scale
MMSE: Mini Mental State Examination
GS: gait speed
BIA: bioelectrical impedance analysis
ASM: appendicular skeletal mass
SD: standard deviation
BMI: body mass index
GH: growth hormone
IGF-1: insulin-like growth factor-1
STAR: sonographic thigh adjustment ratio
PSG: polysomnography
